# Severe visual impairment due to an optic neuropathy and central retinal vein occlusion in a sarcoidosis patient

**DOI:** 10.1186/s12348-020-0198-3

**Published:** 2020-01-31

**Authors:** Miki Hiraoka

**Affiliations:** Department of Ophthalmology, School of Medicine, Sapporo Medical University, Sapporo, Hokkaido 060-8543 Japan

## Abstract

**Background:**

The ophthalmic manifestation of neurosarcoidosis is varied. The complication of optic neuropathy and central retinal vein occlusion (CRVO) is rare in sarcoidosis.

**Case report:**

The patient was a 55-year-old female with systemic sarcoidosis suffering from visual loss as hand motion in her left eye. A fundus examination showed severe optic disc head edema and hyperemia, and a central retinal vein occlusion phenotype including engorgement of all branches of the central retinal vein, dot, and flame-shaped hemorrhages. Brain magnetic resonance imaging (MRI) revealed irregular hypertrophy of the left retrobulbar optic nerve. She received several sets of pulse therapy with intravenous methylprednisolone. Although fundus findings of her left eye and the legion around the left retrobulbar optic nerve showed improvement, the final visual outcome was light perception due to optic nerve atrophy.

**Conclusions:**

Our findings suggest neurosarcoidosis of the unilateral retrobulbar optic nerve can cause compressive optic disc edema and resembles the central retinal vein occlusion (CRVO) phenotype.

## Introduction

Sarcoidosis is an idiopathic multisystem disease characterized by noncaseating granulomatous changes in affected organs. The lung is the most common site of this disease, but the skin, heart, eye, and nervous system can also be involved. The ocular manifestation is mostly uveitis, which is seen in 30–70% of sarcoidosis patients, but rarely in orbit [1, 2].

It has been reported that neurological involvement occurs in 5–26% of sarcoidosis patients [3, 4]. Although any lesion of the nervous system can be affected, those in the cranial nerves and the hypothalamus are affected most frequently. In contrast, impairment of the visual pathway is rare though such disturbances by sarcoidosis can lead to severe visual loss.

In the present report, we describe a sarcoidosis case of unilateral visual deficit with retrobulbar optic neuropathy.

## Case report

The medical records of a patient with unilateral visual impairment affected by sarcoidosis were retrospectively reviewed. The present study protocol was conducted in accordance with the Declaration of Helsinki. After a full explanation of the purpose and protocol for this study was provided to the patient, informed consent was obtained.

A 55-year-old woman developed blurred vision in her both eyes 5 months prior to presentation. She initially visited a separate eye clinic and was diagnosed as having bilateral uveitis. She received topical betamethasone, and her blurred vision reduced in severity. After consultation, she commenced with pulmonary medicine. A chest X-ray demonstrated bilateral hilar lymphadenopathy. Laboratory tests showed an elevated angiotensin-converting enzyme (ACE) in the serum. The specimens from a skin biopsy showed noncaseating granulomas confirming the diagnosis of systemic sarcoidosis. Moreover, for a month prior to the visit, she had suffering from severe vision loss in her left eye.

**Fig. 1:**
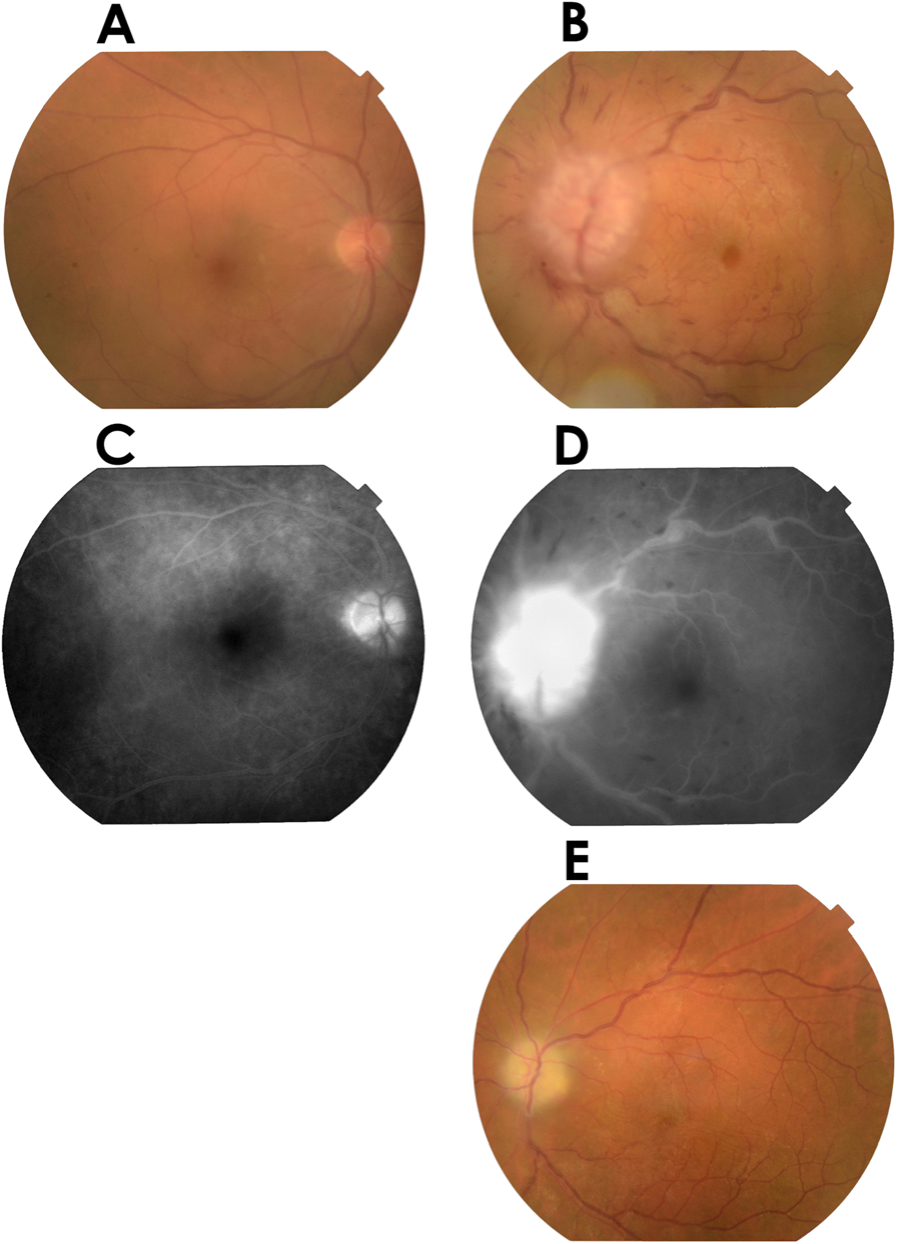
Fundus photograph and fluorescein fundus angiography. Seen in a fundus photograph taken during the initial visit, the right eye shows no abnormality in the post pole (**a**). Left eye shows severe disc edema and hyperemia, and engorgement of all branches of the central retinal vein, dot, and flame-shaped hemorrhages (**b**). Results of fluorescein fundus angiography taken during the initial visit indicate no abnormality in the right eye (**c**). Left eye shows hyperfluorescein in the optic disc (**d**). Seven months later, the optic disc turned pale in color, and retinal hemorrhages disappeared (**e**)

**Fig. 2:**
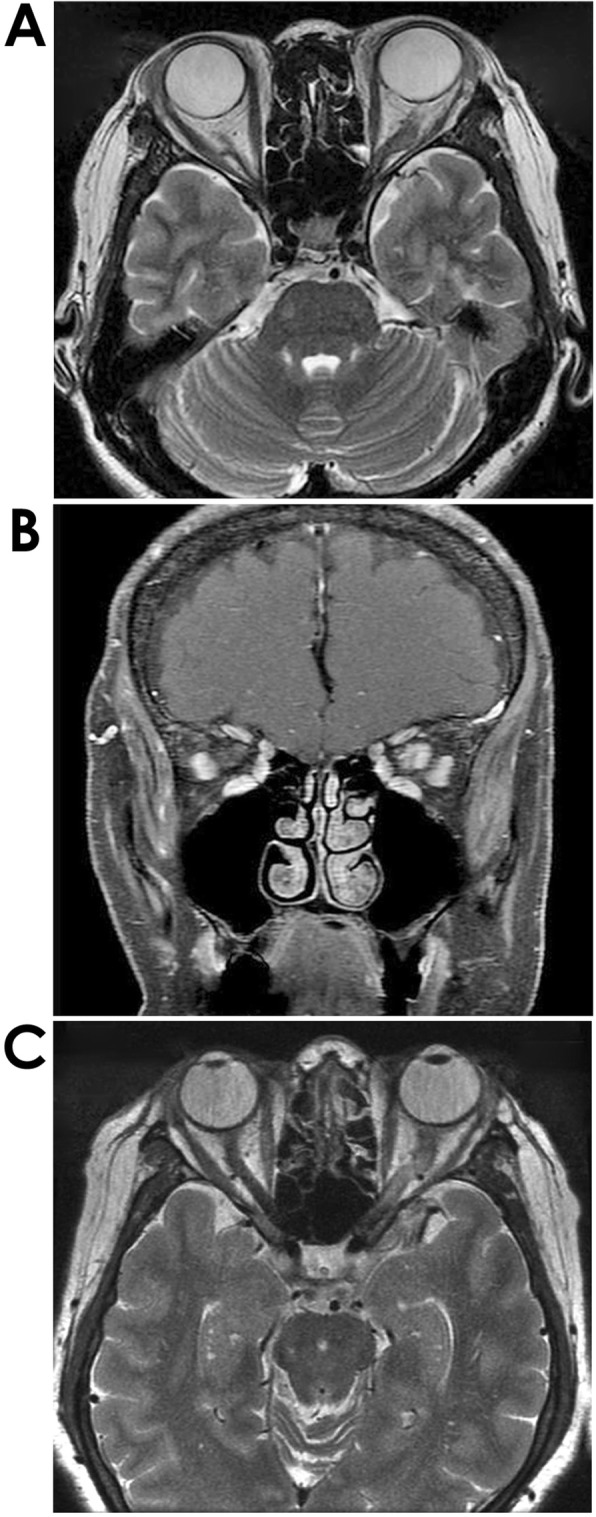
Brain magnetic resonance imaging (MRI). Seen at the initial visit, the axial section revealed irregular hypertrophy of the left retrobulbar optic nerve (**a**). Moreover, the post-contrast T1-weighted image showed enhancement of the left retrobulbar optic nerve (**b**). Five months later, a T2-weighted image indicated that the hypertrophy at the left retrobulbar optic nerve was resolved (**c**)

The initial ophthalmic examination disclosed a best-corrected visual acuity (BCVA) of 20/20 in the right eye and hand motion in the left eye. The intraocular pressure (IOP) was within normal range in both eyes. The relative afferent pupil defect (RAPD) was positive in her left eye. Although a slit-lamp examination showed no cell infiltration in the anterior chamber, gonioscopy indicated tent-shaped peripheral anterior synechia in both eyes. A fundus examination of her left eye demonstrated light vitreous opacity, severe disc edema, and hyperemia and a central retinal vein occlusion phenotype including engorgement of all branches of the central retinal vein, dot, and flame-shaped hemorrhages (Fig. 1b). Her right eye demonstrated retinal perivasculitis in the peripheral area. Fluorescein fundus angiography showed hyperfluorescein in the optic disc, but there was no ischemic area in the retina (Fig. 1d). Brain magnetic resonance imaging (MRI) revealed irregular hypertrophy of the left retrobulbar optic nerve (Fig. 2a). Moreover, the post-contrast T1-weighted image showed enhancement of the retrobulbar optic nerve (Fig. 2b). From these findings, it was presumed that retrobulbar lesion related sarcoidosis caused optic disc edema and central retinal vein occlusion. Against our suggestion, she declined to be hospitalized on that day.

When she was hospitalized a week later, the BCVA of her left eye was reduced to no light perception. Moreover, her left fundus showed increased subretinal hemorrhages. Three sets of pulse therapy with intravenous methylprednisolone (1 g/day for 3 days) following oral prednisolone (25 mg/day) were applied, yielding a positive response. Left eye improved according to the counting fingers scale and optic disc edema, and retinal hemorrhages were reduced. Over the 2 months of oral prednisolone tapering, she complained of ocular pain in her left eye. There was a subsequent relapse of recurrence of optic disc edema and engorgement of retinal veins. One set of pulse therapy with intravenous methylprednisolone (1 g/day for 3 days) following oral prednisolone (25 mg/day) was applied, resulting in an improvement concerning the ocular pain and fundus phenotype. Since then, no relapse has occurred even after tapering the prednisolone. Seven months after the initial visit, optic disc edema, retinal hemorrhages, and engorgement of retinal veins had disappeared, and the optic disc became pale in appearance (Fig. 1e). The BCVA of her left eye was stable at light perception. The hypertrophy at the left retrobulbar optic nerve had resolved when observed under a T2-weighted image scan (Fig. 2c). During the clinical course, there was temporal intraocular pressure elevation and bilateral uveitis that could be maintained by topical betamethasone and antiglaucoma medication.

## Discussion

Neurosarcoidosis occurs in 5–15% of patients with sarcoidosis [3, 5]. Although its legion is usually detected using an MRI, the diagnosis of neurosarcoidosis is challenging due to the difficulty in obtaining biopsy evidence. Thus, it is mainly diagnosed through a clinical presentation in addition to the serum examination, and histological findings in other organs determined to be manifestations of sarcoidosis [6]. According to Koczman et al., 30% of patients showed neuro-ophthalmic manifestations among neurosarcoidosis [7]. The pathological changes were found in 9 out of 19 cases in the optic nerve and in 1 case in the optic chiasm, optic radiations, and the cavernous sinus, respectively. In our case, an MRI examination showed hypertrophy of the left retrobulbar optic nerve. It is believed that sarcoidosis is involved in this hypertrophic area due to its response to systemic corticosteroid therapy. Optic neuropathy is found as a complication in 30–90% patients with neuro-ophthalmic sarcoidosis [7–11]. It includes optic nerve head changes such as edema, hyperemia, atrophy, and granuloma. However, extraocular optic neuropathy is much less common. It is speculated that sarcoid granuloma in the retrobulbar lesion compresses the optic nerve, resulting in severe disc edema and hyperemia in present case.

The retinal vein occlusion is occasionally observed in sarcoidosis [12–15]. According to one report, 14% of patients with sarcoidosis showed branch retinal vein occlusion (BRVO), which is a higher rate than the non-sarcoidosis population [16]. The retinal findings of our case showed the phenotype of impending central retinal vein occlusion. It can be speculated that the compression of the ophthalmic vein in the retrobulbar by sarcoid granuloma caused these fundus changes. There is a separate case report of a patient with unilateral non-ischemic central retinal vein occlusion with similarities to the present case [15]. However, there is no information concerning brain imaging in that case. Rather, visual outcome was mended by systemic corticosteroid therapy. In contrast, vision in present case resulted in a poor outcome. This may due to the long duration between the onset of neuro-ophthalmic sarcoidosis to the introduction of systemic corticosteroid therapy.

The present findings suggest the importance of prompt diagnosis and treatment of neuro-ophthalmic sarcoidosis in the retrobulbar lesion. In cases of sarcoidosis presenting optic neuropathy at the optic disc head or retina vein occlusion, a brain MRI examination might be a good procedure to rule out neuro-ophthalmic sarcoidosis in orbit.

## Data Availability

Data was available in the manuscript.

## Abbreviations

ACE: angiotensin-converting enzyme
BCVA: best-corrected visual acuity
BRVO: branch retinal vein occlusion
CRVO: central retinal vein occlusion
IOP: intraocular pressure
MRI: magnetic resonance imaging
RAPD: relative afferent pupil defect
